# Smoking cigarettes of low nicotine yield does not reduce nicotine intake as expected: a study of nicotine dependency in Japanese males

**DOI:** 10.1186/1471-2458-4-28

**Published:** 2004-07-20

**Authors:** Atsuko Nakazawa, Masako Shigeta, Kotaro Ozasa

**Affiliations:** 1 Division of Health Check-up, Kyoto First Red Cross Hospital 15 Honmachi, Higashiyama-ku, Kyoto, 605-0981, Japan; 2 Department of Epidemiology for Community Health and Medicine, Kyoto Prefectural University of Medicine Graduate School of Medical Science, Kawaramachi Hirokoji, Kamigyo-ku, Kyoto, 602-8566, Japan

## Abstract

**Background:**

Many Japanese believe that low-yield cigarettes are less hazardous than regular cigarettes, and many smokers consume low-yield cigarettes to reduce their risks from smoking. We evaluate the association between actual nicotine intake and brand nicotine yield, and the influence of nicotine dependence on this association.

**Methods:**

The study subjects included 458 Japanese male smokers, aged 51.2 ± 9.9 years, who participated in health check-ups in a hospital in 1998 and 2000. Each subject filled out a self-administered smoking questionnaire and the score of each on the Fagerström Test for Nicotine Dependence was calculated. Urinary cotinine concentration was measured at the time of participation.

**Results:**

The geometric mean of urinary cotinine concentration was 535 ng/mgCr for those who smoked brands with the lowest nicotine (0.1 mg on the package), compared with 1010 ng/mgCr for those who smoked brands with the highest (0.9–2.4 mg, weighted mean of 1.1 mg). Thus, despite the 11-fold ratio of nicotine yield on the packages, the ratio of urinary cotinine level was less than twofold. Both nicotine yield on the package and nicotine dependence significantly increased urinary cotinine concentration, and the negative interaction between them almost attained statistical significance. Cotinine concentration in heavily dependent smokers was consistently high regardless of the nicotine yield of brands.

**Conclusions:**

The nicotine yield of cigarettes measured by machine-smoking does not reliably predict the exposure of smokers. Smokers consuming low-yield nicotine cigarettes did not reduce actual intake of nicotine to the level that might be expected, especially for those heavily dependent on nicotine. Current labeling practices are misleading for the two-third of smokers who are moderately or highly dependent on nicotine.

## Background

'Low-yield nicotine' cigarettes, which have brand names that include 'light,' 'mild,' or similar words, and which have nicotine yields on their packages of 0.8 mg or less, are widely consumed inside and outside Japan, and their market share is increasing. The Tobacco Institute of Japan reported that, in 2001, of the 20 top brands that share 84% of the total cigarettes consumed in Japan, 6.9% had a nicotine yield reported on their package of 0.1 mg, 73.4% had a yield of 0.2–0.8 mg, and 19.7% had a yield of 0.9 mg or higher [1]. The average nicotine yield of these 20 top brands was 0.8 mg, weighted by number of cigarettes consumed [1]. Many smokers would like to avoid the health risks associated with smoking, but not want to quit. These individuals would like to use less hazardous cigarettes or cigarettes that cause less irritation to their throats [2]. In response, the tobacco industry has developed low-yield nicotine brands [3-5].

There have been many studies examining whether low-yield cigarettes are less hazardous than regular brands. For example, in the 1980s and 90s, the actual intake of nicotine [6-9], as well as tar and carbon monoxide [7,9], from smoking low-yield brand cigarettes was similar to that from high-yield brands. More recent studies, which have included ultra-low yield cigarettes (0.1 mg nicotine yield on the package), have shown similar results [10,11]. In addition mortality from lung cancer in the United States has not decreased over the past 30 years, although low-yield brand cigarette increased in market share during that time [12]. Thus consumers of low-yield cigarettes are at a higher health risk than they expected. It has been assumed that high nicotine levels in the blood of smokers of low-yield cigarettes are caused by compensatory behavior due to nicotine dependence. Most of these comparisons have been determined in Western countries. Fewer comparisons have been reported in Japan, and nicotine dependence was partially taken into account during analysis [11].

In Japan, the rate of smoking is still high, being >50% among males[13]. These smokers are likely to consume low-yield cigarettes and to decrease the number of cigarettes consumed in order to reduce the health risks of smoking. For example, a study of smokers in a medical school showed that about 70% of males and 100% of females consumed low-yield nicotine brands [14]. In 1999, some physicians recommended that smokers change to low-yield nicotine cigarettes as the first step toward quitting [15]. In 2000, however, a TV program on scientific issues in Japan reported that low-yield nicotine cigarettes did not reduce the health hazards of smoking [16]. In 2002, the Ministry of Health, Labour and Welfare of Japan published in major Japanese newspapers the findings of a study showing that the nicotine and tar yield of 7 popular cigarette brands in Japan measured by simulating the manner of actual human smoking was larger than that obtained by machines using the Federal Trade Commission (FTC) method [17,18]. Two days later, Japan Tobacco Inc. advertised in these newspapers that the FTC method was authorized throughout the world and that the nicotine yield on the cigarette package was valid for consumers [19]. Thus, issues regarding nicotine yield are less known in Japan than in Western countries. Moreover tobacco companies seem to target young people, especially young women, by intensive advertisement of low-yield cigarette brands [20]. It is important, therefore, to emphasize to smokers the health hazards of low-yield nicotine cigarettes, but evidence in Japanese smokers is still scarce [11]. In the present study, we examined the relationship between nicotine yield and nicotine metabolites excreted in the urine, and the influence of nicotine dependence on this relationship among the Japanese male smokers.

## Methods

The subjects of this study were male smokers who participated in a health check-up at the Kyoto First Red Cross Hospital from July to December in 1998, or from January to February in 2000. The latter subjects were supplementary to the main group, but the two groups exhibited similar demographics. Smokers were recruited using a routine health check-up questionnaire and were defined in this study as those who smoked at least one cigarette per day. Of the 1,579 male participants in the health check-up during the study period, 513 were identified as smokers, and 479 agreed to participate in this study.

Each participant filled out a self-administered questionnaire, which was checked during an interview with a physician. This questionnaire included questions determining score on the Fagerström Test of Nicotine Dependence (FTND) [21]. These included questions on the number of cigarettes smoked per day, time from awaking to the first cigarette, difficulty in refraining from smoking in places where smoking is forbidden, the number of cigarettes smoked during the morning compared with the number smoked during the rest of the day, cigarettes that could not be give up, and smoking for most of the day while ill in bed.

Also, included were questions about the brand(s) of cigarettes smoked, inhalation pattern (deep inhalation, some deep inhalations, or no inhalation), attempt to quit smoking, and stage of behavioral change in the quitting process [22]. Smokers who would continue to smoke during their lifetimes were defined as being on precontemplation-1, and smokers who would continue to smoke for at least one year further but who would quit smoking some day were defined as being on precontemplation-2.

About three quarter of plasma nicotine is converted to cotinine, which is excreted in the urine. The half-life of nicotine is 30 minutes [23] and that of cotinine is about 20 hours [24]. Measurement of cotinine in the plasma, urine is widely used to assess the level of nicotine intake [25]. Therefore, each subject's urinary cotinine concentration was measured. Actual nicotine intake was evaluated from urinary cotinine concentration adjusted for urinary creatinine concentration. Although collection of urine over 24 hours may represent nicotine intake more accurately than a spot urine test, for practical reasons we measured cotinine concentration in the first urine in the morning, as this can reflect smoking from the previous day. Each participant was asked to fast from 21:00 the night before until urine was collected around 9:00 the following morning. The urine samples were frozen at -80°C with in the same day and transported to SRL Laboratory, Hachioji, Tokyo, at which cotinine was measured by gas chromatography [26,27]. For machinery nicotine yield by the FTC method, we used the value indicated on the cigarettes packages.

Statistical analysis was performed using data from 458 male smokers who completed the FTND question and whose urinary cotinine levels were measured. We compared the characteristics of subjects among three groups categorized by machine-measured nicotine yield (0.1 mg, 0.2–0.8 mg, 0.9+ mg). For each group, we calculated mean machine-measured nicotine yield weighted by the number of subjects. Log-transformed data were used for the urinary cotinine concentrations because it was distributed log-normally. Means were compared using Student's t-test or analysis of variance, and proportions were determined using the chi-square test. The effects on urinary cotinine concentration of machine-measured nicotine yield, number of cigarettes consumed per day, and nicotine dependence were analyzed using a regression model, in which urinary cotinine concentration was the dependent variable, and two of the other parameters were independent variables. Main effect and interaction were evaluated by regression coefficients and partial correlation coefficients. The effect of different cigarette brands was also examined. All statistical procedures were performed by SPSS [28]. A P value <0.05 was considered statistically significant.

## Results

Of the 458 subjects, 87 (19.0%) smoked cigarette brands yielding 0.1 mg nicotine, 223 (48.7%) smoked cigarettes of 0.2–0.8 mg, and 148 (32.3%) smoked cigarettes of 0.9+ mg (Table 1). The highest machine-measured nicotine yield for cigarettes consumed by the subjects was 2.4 mg. The weighted mean of brands yielding 0.2–0.8 mg was 0.5 mg, whereas the weighted mean of brands yielding 0.9–2.4 mg nicotine was 1.1 mg. The subjects ranged in age from 23 to 83 years, and the number of cigarettes consumed per day was 1 to 60. Smokers of brands yielding nicotine of 0.1 mg were slightly older than those smoking brands yielding 0.2–0.8 mg and of 0.9–2.4 mg nicotine (p = 0.08). These two groups did not differ with respect to the numbers of cigarettes consumed per day and the FTND score (p = 0.93 and p = 0.20, respectively).

**Table 1:** Characteristics of the subjects by machine-measured nicotine yield of cigarette

Characteristics	Machine-measured nicotine yield (mg/cigarette)			Total	
	0.1	0.2–0.8	0.9–2.4		p
Number of subjects	87	223	148	458	
Mean and SD of mahchine-measured nicotine yield (mg/cigarette)	0.1	0.5 ± 0.2	1.1 ± 0.4	0.6 ± 0.4	
Mean and SD of age	53.6 ± 10.4	50.6 ± 9.34	50.8 ± 10.4	51.2 ± 9.9	0.07
Mean and SD of number of cigarettes per day	23.4 ± 12.2	24.5 ± 10.7	24.4 ± 9.5	24.4 ± 10.6	0.93
Mean and SD of FTND	5.1 ± 2.5	5.4 ± 2.3	5.6 ± 2.0	5.4 ± 2.2	0.21
Number of smokers at each category of FTND					
FTND score 0–3	19 (21.8%)	47 (21.1%)	21 (14.2%)	87 (19.0%)	
FTND score 4–6	40 (46.0%)	100 (44.8%)	78 (52.7%)	218 (47.6%)	0.41
FTND score 7–10	28 (32.2%)	76 (34.1%)	49 (33.1%)	153 (33.4%)	
Number of smokers having attempted to quit	50 (61.0%)	132 (59.5%)	84 (56.8%)	269 (58.9%)	0.73
Number of smokers at each stage of behavioral change in quitting process					
Precontemplation-1	16 (18.6%)	47 (21.3%)	58 (39.7%)	121 (26.7%)	0.001
Precontemplation-2	48 (55.8%)	131 (59.3%)	62 (42.5%)	241 (53.2%)	
Contemplation	20 (23.3%)	33 (14.9%)	23 (15.6%)	76 (16.8%)	
Preparation	2 (2.3%)	10 (4.5%)	3 (2.1%)	15 (3.3%)	
Mean and SD of urinary cotinine concentration (mean-SD, mean+SD*)	535 (1782,160)	770 (1981,299)	1010 (2071,492)	784 (484, 1264)	<0.001

FTND:Fagerström Test for Nicotine Dependence P for difference is examined by analysis of variance or chi-square test * Back-transformation of log-transformed data

Of all subjects, 153 (33.4%) were heavily dependent on nicotine (FTND score>= 7), whereas 87 (19.0%) had low dependence (FTND score< = 3). About 60% of all subjects had attempted to quit, with the proportion similar in the high- and low-nicotine dependent groups (p = 0.73). The stage of behavioral change was different (p = 0.001), however, with smokers of cigarettes yielding 0.1 mg of nicotine being at more advanced stages. The geometric mean of urinary cotinine concentration in all subjects was 784 ng/mg creatinine (Cr), with a distribution of 484 ng/mg Cr (mean-SD) to 1264 ng/mg Cr (mean+SD), as determined by back-transformation of log-transformed data (range; 10–4770 ng/mgCr). The levels differed significantly between the machine-measured nicotine yield groups (p < 0.001).

Urinary cotinine levels did not differ among smokers of individual brands of yielding 0.1 mg of nicotine (p = 0.51 by analysis of variance to adjust for number of cigarettes consumed per day). After integration of similar brands, geometric cotinine concentration means were 686 ng/mgCr for those who smoked American brands and 460 ng/mgCr for those who smoked Japanese brands (p = 0.19). Urinary cotinine concentrations also did not differ among smokers of individual cigarettes brands yielding 0.2–0.8 mg nicotine (p = 0.71, adjusted for number of cigarettes and nicotine yield). The geometric means were 823 ng/mgCr for smokers of Japanese brands and 724 ng/mgCr for smokers of American brands. Mentholated cigarettes were consumed by only 5 subjects and were therefore not examined.

**Figure 1:**
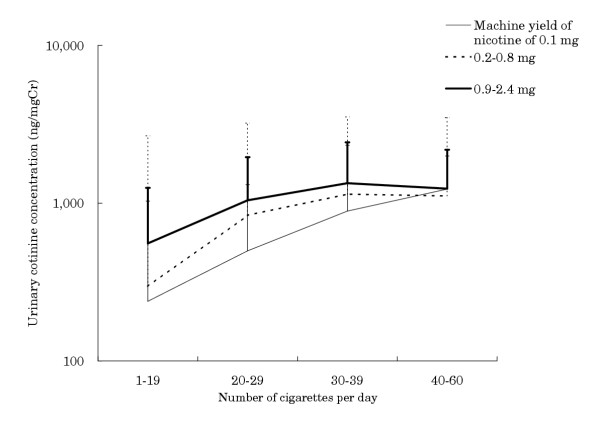
Number of cigarettes per day and urinary cotinine concentration by machine yield of nicotine

When we assayed the relationship between urinary cotinine concentrations and number of cigarettes consumed per day by machine-measured nicotine yield of cigarettes, we found that cotinine concentrations were related to number of cigarettes consumed per day (Figure 1). The correlations were different between machine-measured nicotine yield groups, in that there was a stronger correlation for the low nicotine-yield group. There was some negative interaction between the number of cigarettes smoked and machine-measured nicotine yield (Table 2 upper). When the data restricted with <30 cigarettes consumed per day in which the relationship was assumed to be linear (n = 394), the regression coefficient for machine nicotine yield was 0.834 (p = 0.006), 0.074 (p < 0.001) for number of cigarettes consumed per day, and -0.017 (p = 0.20) for interaction term. Thus, among smokers who consumed a small number of cigarettes, cotinine level of those who smoked high nicotine cigarettes was considerably higher than the level of those who smoked low nicotine cigarettes. In contrast, cotinine level differed little among smokers who consumed 40–60 cigarettes per day, regardless of machine-measured nicotine yield of cigarettes.

**Table 2:** Regression coefficients and partial correlation coefficients for urinary cotinine concentration in a multiple regression model

Variable	Regression coefficients		Partial correlation coefficients	
	B	p	r	p
*Figure 1*				
Intercept	5.270	<0.001		
Nicotine yield by machine	0.762	0.001	0.23	<0.001
Number of cigarettes consumed	0.045	<0.001	0.43	<0.001
Interaction	-0.012	0.16		
*Figure 2*				
Intercept	4.994	<0.001		
Nicotine yield by machine	0.793	0.001	0.18	<0.001
FTND score	0.268	<0.001	0.52	<0.001
Interaction	-0.079	0.057		

FTND: Fagerström Test for Nicotine Dependence

**Figure 2:**
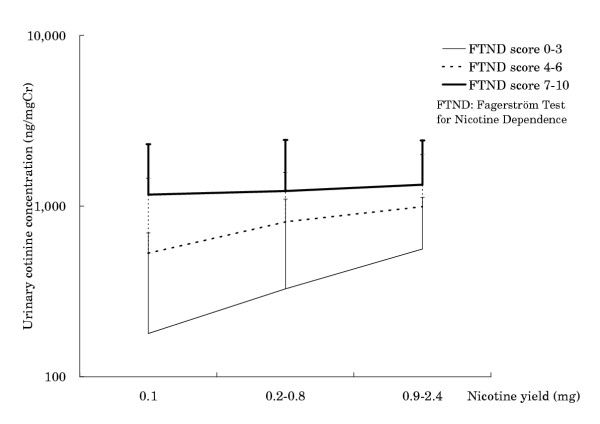
Geometric means of urinary cotinine concentration by nicotine yield and nicotine dependence

When we assayed the relationship between urinary cotinine concentrations and machine-measured nicotine yield of cigarettes by FTND score (Figure 2), we found little difference between machine-measured nicotine yield groups among heavily nicotine dependent smokers, although there was a correlation between urinary cotinine concentration and nicotine yield among smokers with low dependence. According to the regression model, there was an almost significant negative interaction between FTND score and machine-measured nicotine yield (Table 2, lower).

The ratio of mean nicotine yield was 0.45 for cigarettes yielding 0.2–0.8 mg nicotine, and 0.09 for cigarettes yielding 0.1 mg nicotine, compared with the brands yielding 0.9–2.4 mg (Table 3). In contrast, the ratio of mean cotinine concentration was 0.76 for those who smoked cigarettes yielding 0.2–0.8 mg nicotine, and 0.53 for those who smoked cigarettes yielding 0.1 mg nicotine, compared with those who smoked cigarettes yielding 0.9–2.4 mg nicotine. Among heavily dependent smokers the ratios of urinary cotinine concentration were much nearer to 1 (0.92 and 0.85, respectively) than among smokers with low dependence (0.58 and 0.32, respectively).

**Table 3:** Ratios of mean urinary cotinine concentration for nicotine yield by nicotine dependence

Machine measured nicotine yield		Ratio of mean urinary cotinine concentration							
		
		FTND score							
				
Category (mean) (mg)	Ratio of mean nicotine yield								Total
		0–3		4–6		7–10			
0.9–2.4 (1.1)	1	1	(560)	1	(993)	1	(1333)	1	(1010)
0.2–0.8 (0.5)	0.45	0.58	(327)	0.81	(808)	0.92	(1226)	0.76	(770)
0.1 (0.1)	0.09	0.32	(179)	0.53	(529)	0.85	(1138)	0.53	(535)

Numbers in parentheses are geometric means of urinary cotinine concentration (ng/mgCr) FTND:Fagerström Test for Nicotine Dependence

Self-reported inhalation patterns did not influence the average urinary cotinine concentration (p = 0.54) when the variable of inhalation pattern was added to the above model with nicotine yield and FTND score.

## Discussion

In Japan, low nicotine-yield cigarettes seem to be recognized as less hazardous, and smokers likely think that the hazards of smoking are directly proportional to nicotine or tar yield shown on the cigarette packages. This is supported by the results of the present study, which indicate that smokers of low-yield nicotine cigarettes were more advanced behaviorally in wishing to quit. This is additionally supported by circumstantial evidence [1-5,14-18] and by our experience in a check-up clinic, despite the paucity of formal studies of this issue in Japan.

We have shown here that smokers of low nicotine cigarettes did not reduce their actual intake of nicotine to the degree that would be expected from the nicotine yield on the packages. Although smokers of cigarettes yielding 0.1 mg nicotine would be expected to ingest one-eleventh of the nicotine ingested by smokers of cigarettes yielding 0.9–2.4 mg nicotine (average of 1.1 mg), the average urinary cotinine concentration of the former group was more than half that of the latter (535 ng/mg Cr vs. 1010 ng/mg Cr). Moreover, smokers of cigarettes yielding 0.2–0.8 mg nicotine (average of 0.5 mg) had about a 25% decrease in urinary cotinine concentration (770 ng/mg Cr) compared with smokers of cigarettes yielding 0.9–2.4 mg nicotine, despite the two-fold reduction expected from the nicotine yield on the packages. These differences were even smaller in smokers who consumed large numbers of cigarettes per day as well as in smokers with heavy nicotine dependence. The number of cigarettes consumed per day is regarded as a component of nicotine dependence and is included in FTND. There were negative interactions between machine-measured nicotine yield and number of cigarettes consumed per day, and between machine-measured nicotine yield and nicotine dependence. In particular, smokers with heavy nicotine dependence tended to have a high urinary cotinine concentration (about 1200 ng/mgCr) despite differences in machine-measured nicotine yield of cigarettes, which may explain this negative interaction.

In contrast, the actual nicotine intake of smokers who consumed small numbers of cigarettes and smokers with a low level of dependency was more strongly correlated with the machine-measured nicotine yield of the cigarettes they consumed. That is, those who smoked light cigarettes absorbed a smaller amount of nicotine, but, again, the amount absorbed was not equal to the difference in nicotine yields on the packages. These associations are evident in the ratios of means shown in Table 3. Significantly high values of the intercept in the regression models in Table 2 also provide an explanation for the insufficient decrease in urinary cotinine compared with the decrease in nicotine yield on the packages.

We determined the full FTND score in each of our subjects, although some components of FTND, including the number of cigarettes consumed and the time from awakening until the first cigarette, were measured in the previous study of smoking in Japan [11]. It has not been previously reported that smokers with a strong dependency on nicotine showed constantly high levels of urinary cotinine regardless of nicotine yield of the cigarette brands they consumed. Moreover, we recruited a larger number of smokers of cigarettes yielding 0.1 mg nicotine than the previous report [11]. We were thus clearly able to show associations among nicotine dependency, the machine yield of nicotine, and urinary cotinine concentration.

Smokers heavily dependent on nicotine obtained no advantage by smoking low-yield cigarettes. Moreover, they may actually increase their risk due to compensatory behavior, for example, by inhaling more carbon monoxide or other harmful substances contained in cigarette smoke. Our results suggest that tobacco industry advertising may have led these smokers, especially those heavily dependent on nicotine, to underestimate the health risks posed by low-yield cigarettes. This is similar to the results of other studies, which suggested that 'Light' or 'Ultra Light' cigarettes could deliver as much tar and nicotine as 'Regular' cigarettes [6-11].

The compensation mechanisms that may keep blood nicotine at a high level include more puffs per cigarette, greater volume per puff, and greater depth of inhalation, all of which may be conscious or unconscious on the part of smokers. In addition, the filters of low-yield cigarettes are sometimes treated with ammonium to increase absorption of nicotine, thus eliminating the need for deep inhalation [5]. These filters may also be processed to reduce throat irritation, for example in mentholated cigarettes, so that smokers do not realize that are inhaling deeply or frequently [5,29]. This mechanism may increase the inhalation volume, and consequently increase the absorption of carbon monoxide or other harmful substances. In our results, the urinary cotinine concentration did not differ according to self-reported inhalation pattern, suggesting that smokers regulate nicotine intake without being aware of their inhalation patterns. In this study, mentholated cigarettes were not consumed by a sufficient number of subjects for examination. Cigarette brand, however, showed no apparent difference in urinary cotinine level.

Another important mechanism by which smokers of low-yield cigarettes increase their nicotine intake is by blocking the ventilation holes on the filter with their fingers or lips while holding the cigarette and smoking [29]. These holes are made to inspire fresh air and dilute the smoke. During measurement of nicotine yield by the FTC method, however, these holes are not blocked [17].

The tobacco companies have admitted that they have known of the relationship between the nicotine yield reported on the packages and the actual quantities of tobacco smoke components inhaled [30]. Low-yield brands appear to mislead smokers who want to avoid health risks without quitting smoking. Smokers who had not previously considered quitting smoking, however, were found to begin to consider quitting after learning that low-yield cigarettes are processed to make them less irritating and that they did not reduce health risks [31,32]. Most smokers of low-yield brand should be informed of these findings.

The subjects of this study were participants in a so-called 'human dry dock,' a detailed health check-up system for middle-aged and elderly people, which started in Japan in the 1950's and currently enroll about 10 million [33]. Most of them are socioeconomically well off, because each subject pays seven or eight thousand yen ($70–80) on average (up to about forty thousand yen) out-of-pocket for this check-up, and most subjects get annual check-ups. Most participants in this system are health-conscious and have some knowledge about health hazards of smoking. It is likely, therefore, that the subjects of our study are similar with respect to those characteristics, although their occupation, education level, and socioeconomic state were not surveyed. Moreover, in the health check-up associated with this study, many smokers were advised to quit smoking [34], which may explain the relatively low smoking rate of male candidates for this study (32%), compared with an average of 50% or higher in the general community.

Female participants were not examined in this study, mostly because smoking rate of females in our health check-up clinic is less than 10% and some participants refused to answer questions about smoking. However, the advertisement of 'light' cigarettes seems to target young people, especially young women [20], and the smoking rate among young women is increasing in Japan [13]. Thus further studies focused on young people, particularly young females, would provide important information.

In conclusion, we have shown here that the difference in intake of nicotine into a smoker's body was smaller than the difference in machine-measured nicotine yield among cigarette brands. Smokers consuming cigarettes with a low nicotine yield did not reduce actual intake of nicotine to the level that they expected. This was especially true for smokers with heavy nicotine dependence. This result should be emphasized in public health messages to smokers as well as to young people likely to start smoking.

## Abbreviations

FTND: Fagerström Test of Nicotine Dependence, FTC: Federal Trade Commission

## Competing interests

None declared.

## Authors' contributions

AN participated in the design of the study, collected the data, performed the statistical analysis, drafted the manuscript, and was the principal investigator. MNS conceived of the study, participated in its design, and collected the data. KO supervised the data analysis and the manuscript. All authors read and approved the final manuscript.

## Pre-publication history

The pre-publication history for this paper can be accessed here:


